# A novel approach for stabilizing fresh urine by calcium hydroxide addition

**DOI:** 10.1016/j.watres.2016.03.007

**Published:** 2016-05-15

**Authors:** Dyllon G. Randall, Manuel Krähenbühl, Isabell Köpping, Tove A. Larsen, Kai M. Udert

**Affiliations:** Eawag, Swiss Federal Institute of Aquatic Science and Technology, 8600 Dübendorf, Switzerland

**Keywords:** Urine, Source separation, Stabilization of urea, Inhibition of urease, Phosphorus recovery

## Abstract

In this study, we investigated the prevention of enzymatic urea hydrolysis in fresh urine by increasing the pH with calcium hydroxide (Ca(OH)_2_) powder. The amount of Ca(OH)_2_ dissolving in fresh urine depends significantly on the composition of the urine. The different urine compositions used in our simulations showed that between 4.3 and 5.8 g Ca(OH)_2_ dissolved in 1 L of urine at 25 °C. At this temperature, the pH at saturation is 12.5 and is far above the pH of 11, which we identified as the upper limit for enzymatic urea hydrolysis. However, temperature has a strong effect on the saturation pH, with higher values being achieved at lower temperatures. Based on our results, we recommend a dosage of 10 g Ca(OH)_2_ L^−1^ of fresh urine to ensure solid Ca(OH)_2_ always remains in the urine reactor which ensures sufficiently high pH values. Besides providing sufficient Ca(OH)_2_, the temperature has to be kept in a certain range to prevent chemical urea hydrolysis. At temperatures below 14 °C, the saturation pH is higher than 13, which favors chemical urea hydrolysis. We chose a precautionary upper temperature of 40 °C because the rate of chemical urea hydrolysis increases at higher temperatures but this should be confirmed with kinetic studies. By considering the boundaries for pH and temperature developed in this study, urine can be stabilized effectively with Ca(OH)_2_ thereby simplifying later treatment processes or making direct use easier.

## Highlights

• Stabilization of source-separated urine refers to the prevention of urea hydrolysis.
• Calcium hydroxide can be used to stabilize fresh urine.
• Adding Ca(OH)_2_ to fresh urine effectively inhibits enzymatic urea hydrolysis.
• Keeping the temperature between 14 °C and 40 °C limits chemical urea hydrolysis.
• The low solubility of Ca(OH)_2_ allows for passive dosing.

## Graphical abstract

**Figure:**
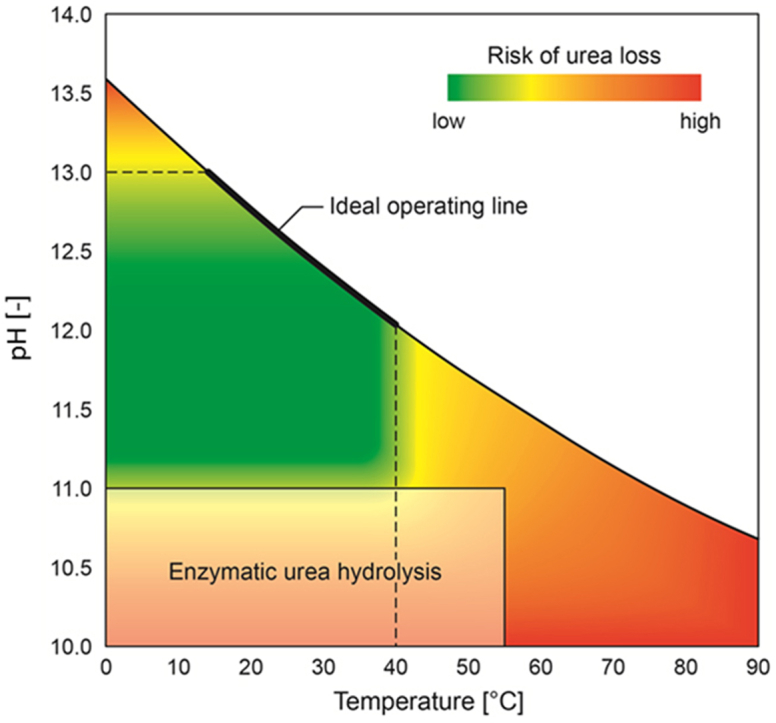

## 1 Introduction

Source separation of human excreta is a resource-efficient alternative to conventional water-borne urban drainage and wastewater treatment (Larsen et al., 2009, Larsen and Gujer, 2001). In fast growing cities, especially in mid- and low-income countries, there is a large potential for source-separating systems (Gounden et al., 2006, Huang et al., 2007, Medilanski et al., 2006), but its implementation is also favorable in developed countries (Larsen et al., 2013). One important aspect of source separation is the recovery of nutrients from urine: it allows the recycling of nutrients to agriculture, prevents environmental pollution and gives the opportunity to recover financial value by selling the nutrients as fertilizer (Udert et al., 2015). To maximize the recovery of nutrients from urine and to prevent malodor, urine has to be stabilized. Stabilization mainly means preventing enzymatic urea hydrolysis. The enzyme urease, which is responsible for enzymatic urea hydrolysis, is ubiquitous in the environment. Consequently, it is only a question of time until urea hydrolysis also occurs in sanitary installations (Udert et al., 2003c). The products of urea hydrolysis are free volatile ammonia and carbon dioxide. When ammonia volatilizes from urine, the corresponding amount of nitrogen is lost for fertilization and at the same time causes environmental pollution. Volatilization can take place at different steps in urine handling: during storage, transport, application, treatment and especially during volume reduction, e.g. by evaporation.

Different methods have been investigated to prevent volatilization of ammonia, either by inhibiting urea hydrolysis or by converting free ammonia to non-volatile ammonium. Enzymatic urea hydrolysis can be prevented by acid addition (Hellström et al., 1999), the addition of urease inhibitors (Adams et al., 2012) or by electrochemical treatment (Ikematsu et al., 2007). If urea hydrolysis cannot be prevented, a pH decrease is needed to shift the ammonia-ammonium equilibrium towards the non-volatile ammonium. This can be achieved either by direct acid addition (Ek et al., 2006) or by nitrification (Udert et al., 2003a). Although these methods are effective, they all have flaws, especially for on-site application: the addition of acid is potentially dangerous, requires pumping equipment and exact dosing; electrochemical treatment only allows for short-term inhibition of enzymatic urea hydrolysis and does not prevent later contact with urease-active microorganisms; finally, partial nitrification is a complex biological process that requires process regulation–at least at the present level of development (Fumasoli et al., 2016).

An alternative and potentially more elegant stabilization method would be the inhibition of enzymatic urea hydrolysis at high pH values. Urease activity is negatively affected not only by a low pH but also by a high pH. The pH optima for bacterial urease have been reported to be in a range of 6.8–8.7 (Mobley and Hausinger, 1989). In contrast to strong acids, several strong bases are available as basic salts such as calcium and magnesium oxides and hydroxides. These basic salts are advantageous for several reasons: firstly, they are commonly used to enhance soil quality–primarily in order to increase the pH of acidic soils, but also to provide essential plant nutrients such as calcium and magnesium (Shaul, 2002). Secondly, calcium and magnesium form phosphate minerals at high pH values, allowing the separation of phosphates. Finally, there is a good possibility that the high pH of the solution will reduce the number of pathogens considerably, for example viruses and helminthes (Eriksen et al., 1996, Pancorbo et al., 1988). However, urea is not only degraded by the enzyme urease. At high pH values and high temperatures, urea also decomposes chemically (Warner, 1942) into ammonia and carbon dioxide. This process has to be considered when stabilizing urine at high pH values.

In this study we assessed the feasibility of preventing enzymatic urea hydrolysis in fresh urine by three common calcium compounds: calcium oxide (CaO), calcium hydroxide (Ca(OH)_2_) and calcium carbonate (CaCO_3_). We chose these compounds because they are cheap (US$ 0.08 kg^−1^ for Ca(OH)_2_) (Muster et al., 2013) and because CaO and Ca(OH)_2_ are commonly used in sanitation for disinfection purposes (Ronteltap et al., 2014). Another benefit of Ca(OH)_2_ is that calcium phosphate precipitates during the stabilization process which can be recovered and used for fertilizer production (Rittmann et al., 2011). We also developed a preliminary design chart showing conditions where urea loss will likely be minimized.

## 2 Materials and methods

### 2.1 Simulation of chemical speciation

The software PHREEQC version 3.1.7.9213 (Parkhurst and Appelo, 1999) was used to simulate the speciation of chemical compounds at thermodynamic equilibrium. The components used for the chemical speciation calculations are shown in Table 1. Simulations were conducted for different urine compositions (Table 2). For ionic strength corrections, we used the Pitzer approach implemented in a database described in (Wächter et al. in prep.). The composition of fresh urine was based on results from real human urine. Urine samples U1, U2, U3 and U4 were characterized in this study while the composition of urine samples U5 (Udert et al., 2003b) and U6 (Udert et al., 2006) were obtained from literature. All the compounds in the lower part of Table 2 were used in the simulations. Acetate was used as a proxy for chemical oxygen demand (COD), under the assumption that 1 g COD is equivalent to 1.08 g of acetate (Grady et al., 1999).

### 2.2 Urine solutions

For all experiments described in this paper, fresh male and female human urine was collected in glass bottles (10 persons, age 25–50). For longer experiments, the urine samples were stored at 4 °C before use. Only urine with a pH lower than 6.9 was used, because higher pH values were an indication of urea hydrolysis and therefore contamination with urease-active microorganisms (Udert et al., 2003b). Urine samples U1a and U4 (Table 2) depict average compositions of the fresh urine used in the experiments for this study.

### 2.3 Dissolution of Ca(OH)_2_ in fresh urine

A dosage of 5 g Ca(OH)_2_ (102047 Calcium hydroxide, Merck, Darmstadt, Germany) was added to 1 L of fresh urine to investigate the dissolution. The dosage was based on results from preliminary PHREEQC simulations, which showed that 4.3 g Ca(OH)_2_ is the maximum solubility for the specific urine composition (U1) used in the experiments at 25 °C. During the dissolution of Ca(OH)_2_ in fresh urine, pH was measured with 5 s intervals (SenTix 41, WTW, Weilheim, Germany). The initial pH of the urine was 6.63.

### 2.4 Ideal pH range for inhibition of enzymatic urea hydrolysis

For the experiments to determine the ideal pH range for the inhibition of enzymatic urea hydrolysis, nine 250 mL glass bottles (Schott, borosilicate glass 3.3, Mainz, Germany) were filled with 250 mL unfiltered urine (U4). The bottles were washed thoroughly with hot water and detergents to minimize the presence of urease. The bottles were also stored closed with lids. To all bottles, 37 μL urease (116493 Urease, Merck, Darmstadt, Germany) was added to ensure a urea hydrolysis rate of 3000 g N m^−3^ d^−1^ which is typical for urine storage tanks (Udert et al., 2003b). The pH of the eight bottles was adjusted with 10 mol L^−1^ NaOH (Merck, Darmstadt, Germany) at different pH levels between 9.0 and 12.5 in increments of 0.5 pH units. In one bottle the pH was not adjusted and served as a blank. All bottles were placed in the dark and agitated at 150 rpm using a plate shaker (SM-30, Edmund Bühler GmbH, Hechingen, Germany). The duration of the experiment was 10 days and the ammonium concentration was measured and the pH recorded. For analysis, each bottle was placed on a magnetic stirrer at 500 rpm and a 1 mL unfiltered sample was taken once the pH was stable. The pH of each bottle was controlled daily and readjusted with 10 mol L^−1^ NaOH, if required. The pH probe was calibrated weekly with pH 4 and pH 7 buffer solutions.

### 2.5 Long-term stabilization of fresh urine with Ca(OH)_2_

The long-term behavior of urine dosed with Ca(OH)_2_ was investigated for the addition of varying amounts of Ca(OH)_2_ to 1 L of fresh urine (U1a): 2.5 g, 5 g and 10 g. Urease (116493 Urease, Merck, Darmstadt, Germany) was added to the batches in order to obtain a urea hydrolysis rate of 3000 g N m^−3^ d^−1^. Two control experiments were also conducted, one without adding either urease or Ca(OH)_2_ and one with urease but no Ca(OH)_2_. All batches were covered in order to reduce ammonia volatilization and continuously stirred. Total ammonia concentrations were regularly measured (cuvette test for ammonium, Hach-Lange, Düsseldorf, Germany) and pH (340, WTW, Weilheim, Germany) was measured whenever a sample was taken. The stabilization experiment was conducted for 27 days.

### 2.6 Stabilization of consecutive batches of fresh urine with Ca(OH)_2_

An initial deposit of 5 g Ca(OH)_2_ was provided followed by additional batches of fresh urine (U1a) (250 ml) after 24, 48 and 72 h. The mixture was continuously stirred magnetically. All portions of fresh urine, which were added to the reactor, had a pH value below 6.9. During the experiment, the batch was covered to avoid volatilization of ammonia and the pH was measured shortly before and after adding a new portion of fresh urine.

### 2.7 Analytical methods

Concentrations of dissolved compounds were determined with ion chromatography (IC 881 Compact IC pro, Metrohm, Zofingen, Switzerland) and inductively coupled plasma-optical emission spectrometry (ICP-OES, Ciros, Spectro Analytical Instruments, Kleve, Germany). The standard deviation for all wet chemical measurements was 5% or less.

### 2.8 Analysis of precipitates

Urine and Ca(OH)_2_ were mixed with a magnetic stirrer, left overnight without stirring and centrifuged to separate solids from the liquid fraction (10 min at 4000 rpm). The separated solids were dried under a fume hood for several days. X-ray diffractrometry (XRD) (Power X-ray diffractometer AXS D8 Advance, Bruker, Coventry, UK) was used to determine the composition of the precipitates.

## 3 Results

### 3.1 Simulation of Ca(OH)_2_ solubility in fresh urine

**Fig. 1:**
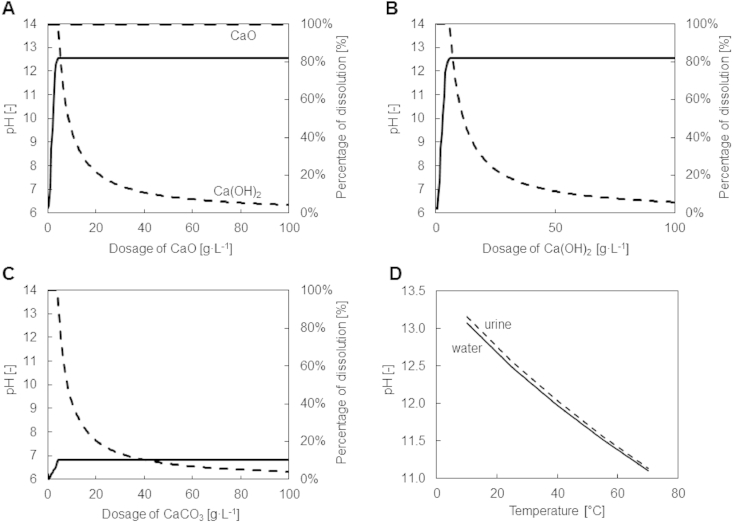
Simulated pH () and percentage of dissolution () for the addition of CaO (A), Ca(OH)_2_ (B) and CaCO_3_ (C) to fresh urine at 25 °C and saturation pH of Ca(OH)_2_ in urine () and water () as a function of temperature (D). When CaO is dissolved in water, it converts to Ca(OH)_2_. For this reason, the dissolution of CaO as well as the dissolution of Ca(OH)_2_ are depicted in A.

In Fig. 1A–C the simulated percentage dissolution and the resulting pH at 25 °C are shown for different dosage concentrations of CaO, Ca(OH)_2_ and CaCO_3_ added to fresh urine. Calcium oxide is highly soluble, but immediately transforms to Ca(OH)_2_, giving rise to identical results as for Ca(OH)_2_. Both Ca(OH)_2_ and CaCO_3_ are sparingly soluble: a saturated solution is rapidly achieved for all of the three minerals: at 3.5 g CaO L^−1^, 4.3 g Ca(OH)_2_ L^−1^ and 2.0 g CaCO_3_ L^−1^. However, the addition of CaCO_3_ only leads to a pH of 7.2, whereas the addition of CaO and Ca(OH)_2_ results in a pH of 12.5. As typical pH optima for urease are between 6.8 and 8.7 (Mobley and Hausinger, 1989), CaCO_3_ dosage is unsuitable for the inhibition of enzymatic urea hydrolysis.

Since CaCO_3_ is not suitable for urease inhibition and CaO is immediately transformed into Ca(OH)_2_, the theoretical saturation pH as a function of temperature was only assessed for the latter (Fig. 1D). The saturation pH decreases as the temperature increases. The urine curve represents the saturation pH values for all the compositions given in Table 2. The saturation pH values were the same even though the compositions differed substantially. In fact, even in pure water, the saturation pH values of Ca(OH)_2_ is similar to that of urine (Fig. 1D).

**Fig. 2:**
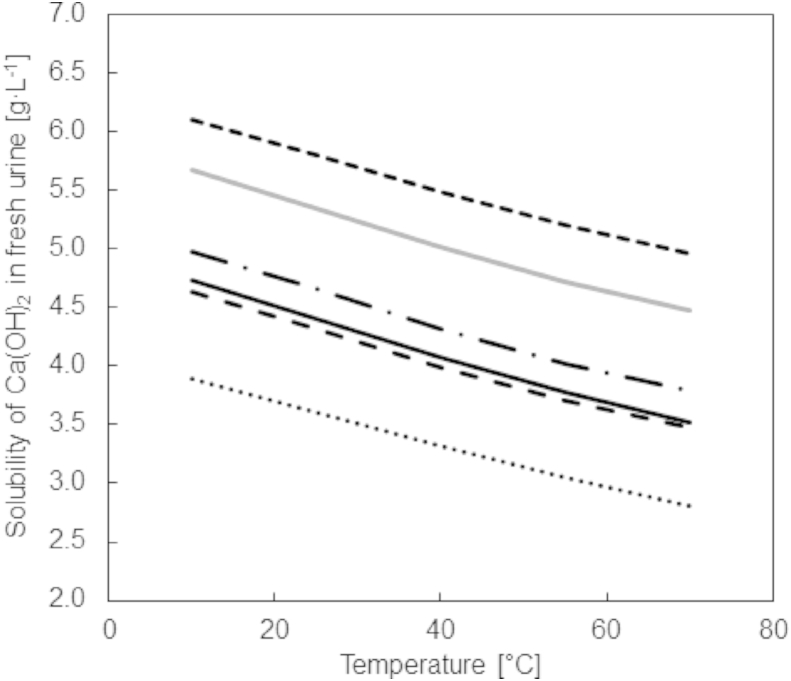
Simulated amount of Ca(OH)_2_ dissolving in different urine compositions: U1 (), U2 (), U3 (), U4 (), U5 () and U6 (). The compositions are given in Table 2.

Fig. 2 shows the amount of Ca(OH)_2_ that dissolves in different urine compositions as a function of temperature and varies significantly depending on the urine composition and, to a lesser extent, the temperature. For example, the composition used in the experimental work of this study (U1) resulted in a simulated value of 4.3 g Ca(OH)_2_ L^−1^ dissolving at 25 °C while 5.80 g Ca(OH)_2_ L^−1^ dissolved for composition U5 at 25 °C. The simulation results (Fig. 2) also illustrate that Ca(OH)_2_ has an inverse solubility which means that less solid dissolves at higher temperatures.

### 3.2 Dissolution of Ca(OH)_2_ in fresh urine

The experimental addition of 5 g Ca(OH)_2_ L^−1^ of urine at 25 °C led to comparable results for the simulated and measured composition of stabilized urine (Table 2): dissolved phosphate and magnesium were nearly completely removed from the liquid phase, whereas the concentration of dissolved calcium increased. All other parameters, with the exception of pH, are only marginally affected by the addition of Ca(OH)_2_.

**Fig. 3:**
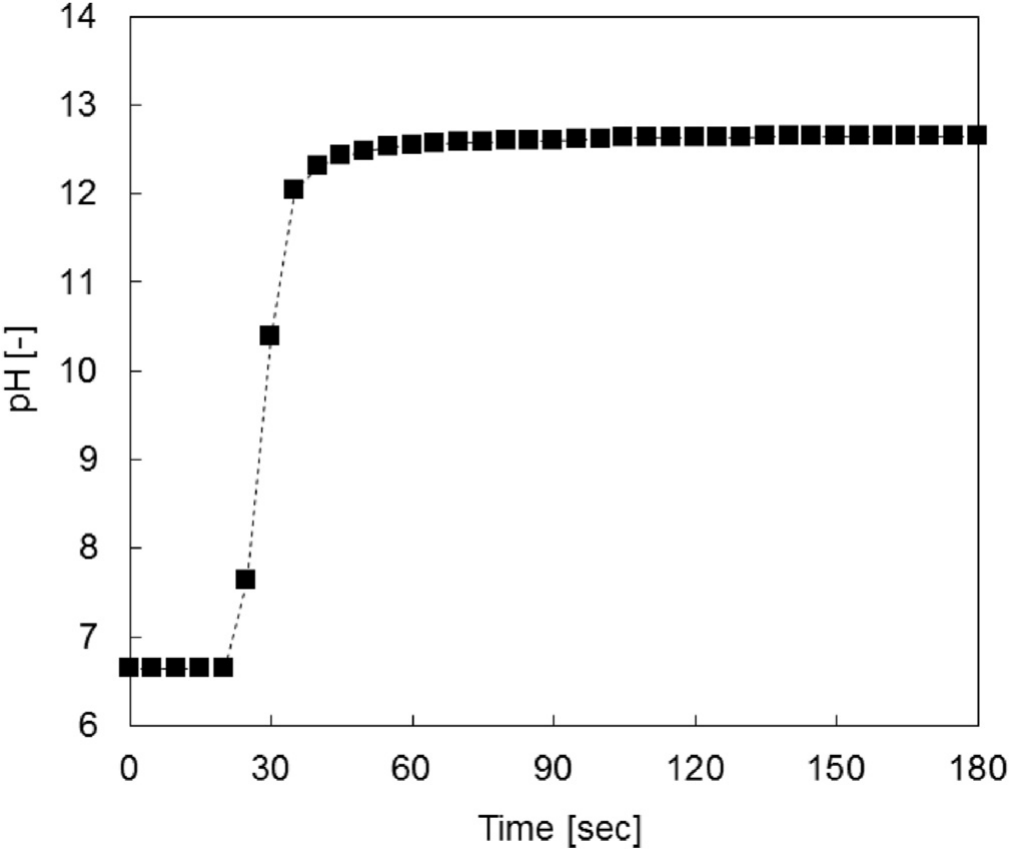
Experimentally observed pH increase after the addition of 5 g Ca(OH)_2_ to 1 L of fresh urine.

**Fig. 4:**
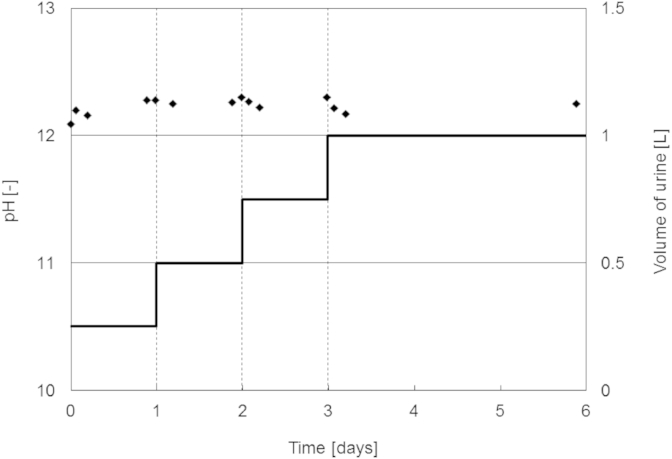
Course of the pH value (♦) during consecutive addition of fresh urine to initial deposits of Ca(OH)_2_. Every dotted vertical line refers to the addition of fresh urine to the Ca(OH)_2_ deposit. The bold line depicts the cumulative volume of fresh urine. The initial dosage of Ca(OH)_2_ was 5 g.

In the experiments with 5 g Ca(OH)_2_, a stable high pH value was reached within less than 1 min (Fig. 3). A Ca(OH)_2_ deposit could keep the pH of consecutively added fresh urine between 12 and 12.5 during the experimental duration of 6 days (Fig. 4). For all laboratory experiments, the measured pH increase caused by the addition of Ca(OH)_2_ was in line with the results of the computer simulations: a pH around 12.5 was found for dosages of at least 5 g Ca(OH)_2_ L^−1^

### 3.3 Experimentally determined urine stability at high pH values

**Fig. 5:**
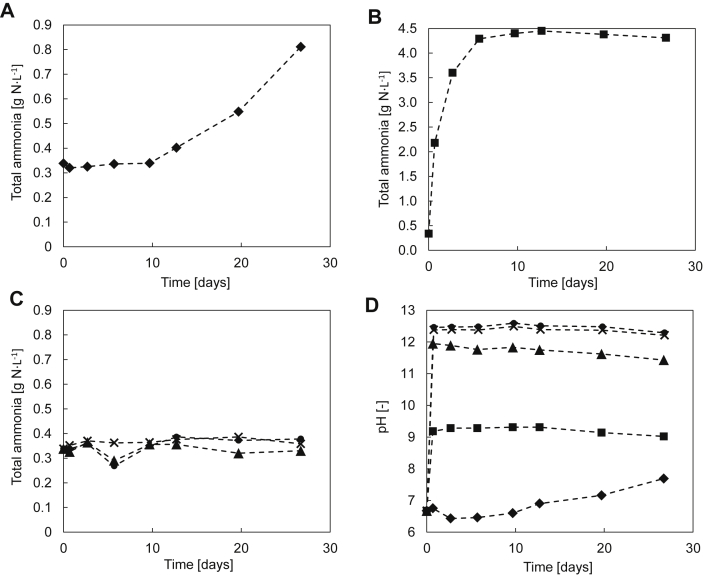
Experimentally observed total ammonia concentrations (A, B and C) and pH (D) during the long-term stabilization of fresh urine with Ca(OH)_2_: Pure fresh urine (♦), fresh urine spiked with urease (■), fresh urine spiked with urease and 2.5 g Ca(OH)_2_ L^−1^(▴), fresh urine spiked with urease and 5 g Ca(OH)_2_ L^−1^ (×) and fresh urine spiked with urease and 10 g Ca(OH)_2_ L^−1^ (●). The initial conditions of all urine batches at t = 0 were pH 6.67 and 338 mg N L^−1^ total ammonia.

Calcium hydroxide addition can prevent urea hydrolysis for a long period, in our experiments, 27 days (Fig. 5). In the case of pure fresh urine without the addition of urease or Ca(OH)_2_, there is only a slow increase in ammonium leading to a doubling of the ammonium concentration after 27 days, which implies that the urine contained few urease-active microorganisms (Fig. 5A). When the untreated urine was spiked with urease, the ammonium concentration increased rapidly: within the first 10 days of the experiment, the concentration increased to about 4300 mg N L^−1^ (Fig. 5B), which is more than tenfold of the initial concentration (340 mg N L^−1^). As depicted in Fig. 5C, the ammonium concentration of urine spiked with Ca(OH)_2_ remained within the initial range. Fig. 5D shows that the pH in fresh urine spiked with urease reaches a value of 9.3 within the first day. In contrast, the pH of pure fresh urine slowly increased to 7.7 within the 27 days of the experiment. For urine spiked with Ca(OH)_2,_ a pH value of 12.4 was reached with 5 g Ca(OH)_2_ L^−1^ or more. However, with an addition of 2.5 g Ca(OH)_2_ L^−1^, the pH value stayed at 11.9, which indicates that the solution was not saturated with Ca(OH)_2_.

**Fig. 6:**
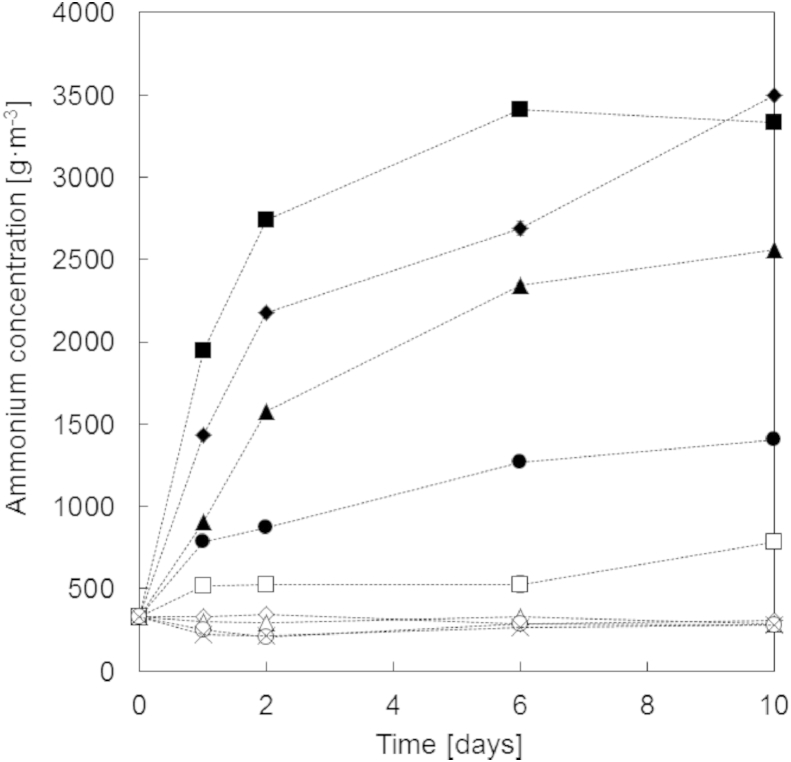
Ammonium concentration at different solution pH values for urine composition U4 (see Table 2): pH = 9 (♦), pH = 9.5 (▴), pH = 10 (●), pH = 10.5 (□), pH = 11 (◇), pH = 11.5 (△), pH = 12.0 (○), pH = 12.5 (×) and the reference (■).

A set of experiments using urine composition U4 and fixed pH values was conducted in order to determine, how far the pH value has to be increased to inhibit enzymatic urea hydrolysis. The production of total ammonia during ten days was used as a measure of the extent of enzymatic urea hydrolysis (Fig. 6). The total ammonia concentration did not change substantially, when the pH value was 11 or higher. At lower pH values, the production of ammonia increased with decreasing pH values. The pH adjustments were done with NaOH and not calcium compounds for these experiments.

### 3.4 Calculation of non-enzymatic urea hydrolysis

**Fig. 7:**
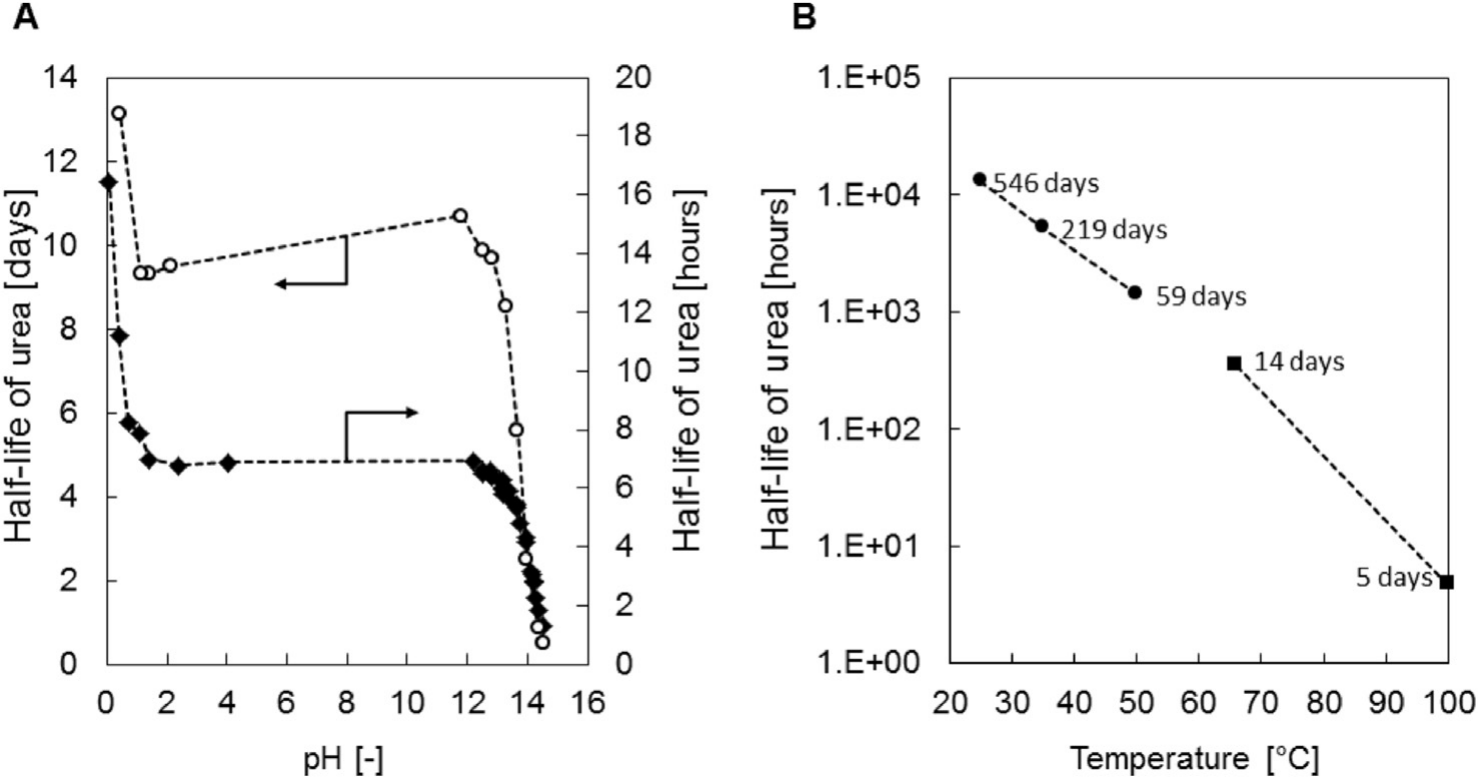
(A) Half-life of urea for different pH levels based on the non-enzymatic hydrolysis postulated by Warner (1942) at 66 °C (o) in days and 100 °C (◆) in hours. (B) Stability of urea affected by non-enzymatic hydrolysis: The values at 66 °C and 100 °C (■) are directly derived from Warner (1942), while the data at 25 °C, 35 °C and 50 °C (●) are derived from (Chin and Kroontje, 1963).

Other researchers have shown that chemical urea hydrolysis strongly depends on pH (Warner, 1942) and temperature (Chin and Kroontje, 1963, Warner, 1942) as shown in Fig. 7. According to the literature data, the half-life of urea is in the range of days below 66 °C, whereas at 100 °C, the half-life is reduced to hours (Fig. 7A and B). The pH value also influences urea hydrolysis, but only at the extreme ends that is below pH 2 and above pH 12 (Fig. 7A). At pH values higher than 12, the half-life is decreasing, though a significant reduction of the half-life is only observed, when the pH values rise above 13. Fig. 7B shows the impact of temperature on urea, where an increased chemical hydrolysis is favored by increasing temperature.

### 3.5 Simulation of phosphate precipitation

**Fig. 8:**
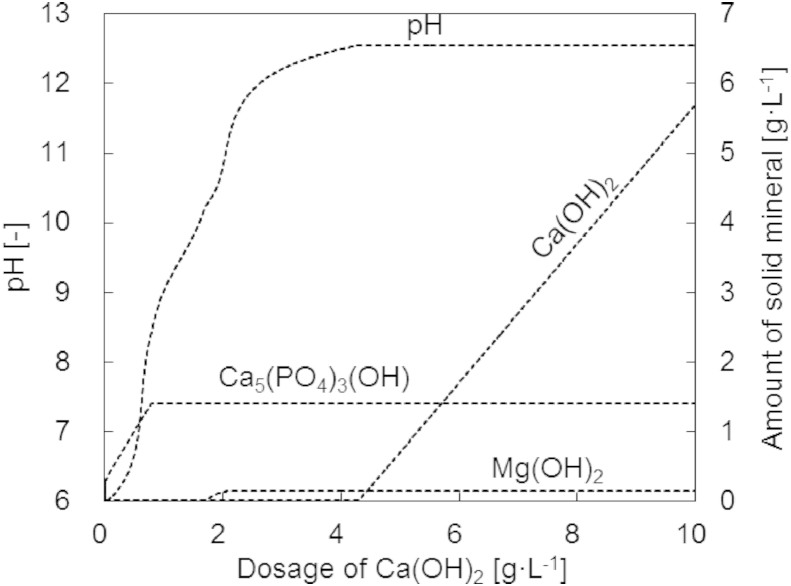
Simulated pH and presence of solid mineral phases for varying dosages of Ca(OH)_2_ to fresh urine at 25 °C: Mg(OH)_2_, Ca(OH)_2_ and Ca_5_(PO_4_)_3_OH.

Our experimental results showed that the addition of Ca(OH)_2_ caused complete precipitation of phosphate and magnesium as indicated by the decrease in concentration (Table 2). It is known that calcium phosphate compounds such as apatite precipitate at high pH values (Nriagu et al., 1984). However, XRD analysis of the precipitates revealed only the presence of the calcium compounds Ca(OH)_2_ and CaCO_3_, and not of any crystalline calcium phosphate minerals. Also, no struvite pattern was found, although struvite typically precipitates from urine at elevated pH values (Udert et al., 2003a). Computer simulations of the chemical speciation indicate that hydroxyapatite (Ca_5_(PO_4_)_3_(OH)) should be present at thermodynamic equilibrium (Fig. 8). However, amorphous calcium phosphate (usually not detected by XRD analysis) is often the first compound to precipitate (Nancollas and Zhang, 1994) and it takes some time to transform to Ca_5_(PO_4_)_3_(OH). According to the computer simulations, the magnesium found in urine at these conditions precipitates as Mg(OH)_2_. For dosages of Ca(OH)_2_ around 4.3 g and higher, an increase in Ca(OH)_2_ can be observed due to incomplete dissolution of the compound.

## 4 Discussion

### 4.1 Urine stabilization with Ca(OH)_2_

**Fig. 9:**
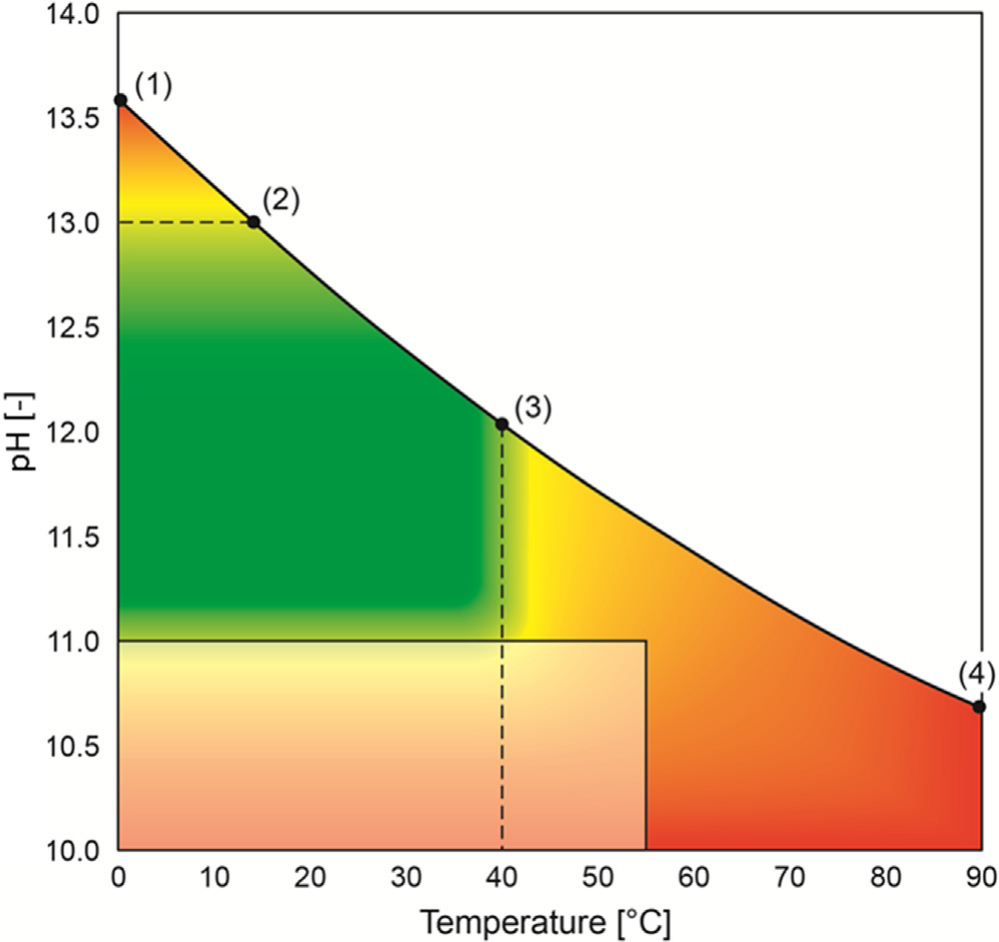
Design chart showing areas where negligible urea loss occurs (green), enzymatic urea hydrolysis occurs (bottom rectangular region, between pH 10 to 11 and temperature 0–55 °C) and where chemical urea decomposition is more abundant (yellow–orange–red). Red being the region where the greatest urea loss will likely occur. The dotted lines represent precautionary limits for chemical urea hydrolysis, while the straight white lines approximate the limits for enzymatic urea hydrolysis. The saturation pH curve for Ca(OH)_2_ is represented by line 1–2 – 3–4.

We have developed a tentative design chart (Fig. 9) that can be used to determine the ideal operating conditions with respect to pH and temperature for the stabilization of urine with Ca(OH)_2_. The solubility of Ca(OH)_2_ defines the upper boundary of the design chart. The saturation pH does not depend on the composition of urine (Fig. 1D) but the amount of Ca(OH)_2_ required to create a saturated solution does depend on the composition of the urine (Fig. 2). For example, the experimental results showed that a dosage of 5 g Ca(OH)_2_ was enough to keep urine composition U1 stabilized since the saturation concentration was 4.3 g Ca(OH)_2_ L^−1^ of urine. However, a dosage of 5 g L^−1^ of urine would not saturate urine compositions U5 and U6. The complex aqueous chemistry and variability in compositions between urine samples results in different Ca(OH)_2_ solubilities in fresh urine. For this reason, we recommend a dosage of 10 g Ca(OH)_2_ L^−1^ of fresh urine because this will effectively stabilize a wide range of urine solutions with different compositions while still retaining residual solid Ca(OH)_2_ in solution to ensure saturation (Fig. 4).

The design chart (Fig. 9) also shows that for temperatures below 14 °C, a saturation pH of 13 (point (2)) or higher is obtained. At these low temperatures the rate of chemical urea hydrolysis will be slow but the excessively high pH values will increase the rate significantly. As a first guess, we suggest a minimum temperature of 14 °C as a limit. A more accurate limit will have to be determined with specific kinetic experiments.

The shaded green region in the design chart is a region where urea loss is low because both enzymatic urea hydrolysis and chemical urea hydrolysis are limited. A saturated Ca(OH)_2_ solution would operate along line 2–3 which would be the ideal conditions of operation. Technically, operation could also occur below this line, in the green area, but operation in this region would be challenging because it would require active dosing of Ca(OH)_2_ or another alkaline chemical using dosing pumps or solid dosing devices and additional equipment. Our approach of passive dosing with a fixed deposit of Ca(OH)_2_ powder, in excess of that required for saturation, is much easier because no active pH control is then required. We also have to ensure that Ca(OH)_2_ is added to fresh urine as soon as possible, ideally in a stabilisation tank below the toilet or urinal so that the piping between the user interface and the stabilization tank is short and the retention time is too low for substantial urea hydrolysis to occur.

The transparent rectangular region demarks conditions which favor enzymatic urea hydrolysis. Based on our finding, the pH value in fresh urine has to be 11 or higher to suppress urease activity at a temperature of 25 °C. At higher temperatures and pH values below 11 there is a transition from enzymatic to chemical urea hydrolysis. Operating above a temperature of 55 °C inactivates the enzyme urease (Lencki et al., 1992) therefore chemical urea hydrolysis is the only reason for urea loss above this temperature.

Excessively high temperatures have to be avoided because this promotes the chemical hydrolysis of urea. We have introduced a tentative and conservative border for safe urea stabilization at 40 °C. The exact upper temperature limit has to be determined as a function of hydrolysis kinetics, duration of urine storage, acceptable ammonia losses and the desirable safety factor. Further research into the kinetic rates of chemical urea hydrolysis in urine is therefore needed as this will help to better define this temperature boundary.

### 4.2 Alternative calcium salts for dosing

Calcium carbonate dissolution will cause a pH increase in fresh urine, but the resulting pH is too low (less than 7.2) to inhibit urease activity. Therefore, CaCO_3_ is not suitable for urine stabilization. Calcium oxide is also not suited for urine stabilization but for other reasons. The addition of CaO to fresh urine leads to pH levels around 12, which is sufficient for suppressing the enzymatic conversion of urea to ammonia under ambient temperatures. However, when CaO is dissolved in aqueous solutions, such as urine, a vigorous release of heat of 1.14 MJ kg^−1^ (Girovich, 1996) occurs, which can lead to a temperature increase up to the boiling point. This temperature increase can be dangerous and can also lead to chemical urea hydrolysis. Therefore, we do not recommend using CaO for the prevention of urea hydrolysis in urine.

### 4.3 Advantages for inclusion in a nutrient recovery process

The stabilization of fresh urine with Ca(OH)_2_ has a number of advantages, especially when included in a process for nutrient recovery. Three of these advantages are described in the following sections.

#### 4.3.1 Ammonia stabilization for subsequent water removal

The stabilization of fresh urine strongly limits the amount of ammonia that will volatilize thus allowing for subsequent water removal techniques to be used. The reduction of the stabilized urine volume will reduce transport costs while the increasing salinity of the concentrated liquid will further reduce urease activity (Hotta and Funamizu, 2008).

One method to reduce the volume of urine is by vapor pressure distillation which Udert and Wächter (2012) showed was effective for nitrified urine. In the case of urine stabilized by Ca(OH)_2,_ this method would not be applicable due to elevated temperatures during distillation and the subsequent loss of urea by chemical urea hydrolysis. Three possible alternative processes for volume reduction are electrodialysis, reverse osmosis or freeze/thaw concentration. However, besides urea stability, the energy demand will be decisive for the choice of the process (Udert and Wächter, 2012). Another alternative process is passive evaporation at ambient temperatures (Bethune et al., 2015) which could be an effective method because the ambient temperatures would be ideal for preventing chemical urea hydrolysis, provided a saturated Ca(OH)_2_ solution is maintained to prevent enzymatic urea hydrolysis. However, any post stabilisation process, such as evaporation, should keep the temperature and pH within the recommended range during storage and transport. Consequently, any impacts on pH and temperature must be considered (in addition to energy requirements) when selecting downstream processing and distribution of stabilised urine. Moreover, other factors that should be considered include the ratio of monovalent to divalent cations in the stabilised urine which will affect soil sodicity and salinisation potential. Therefore, downstream processing would need to be evaluated on a case-by-case basis.

#### 4.3.2 Potential targeted phosphate removal

The addition of Ca(OH)_2_ does not only enable the preservation of urea, but also the separate recovery of phosphate by precipitation as calcium phosphate. Dosing a calcium salt for phosphate recovery by calcium phosphate precipitation is only economical if fresh urine with a high pH is used. If calcium is added to stored urine, that means after urea was hydrolyzed, calcium carbonate will precipitate instead of calcium phosphate.

To recover phosphate from stored urine, struvite can be precipitated by dosing a magnesium compound (Etter et al., 2011). However, a magnesium salt such as Mg(OH)_2_ would not stabilize fresh urine even though it would result in struvite precipitation because simulation results (not shown) indicate the saturation pH would not exceed 10.

In addition, the use of Ca(OH)_2_ over Mg salts has several advantages. The use of calcium phosphate in the phosphate industry is more likely compared to struvite (Driver et al., 1999). Calcium phosphate is less sensitive to heat, whereas inadequate drying, storing or transporting of struvite at temperatures higher than 60 °C can cause its decomposition and the loss of ammonia (Sarkar, 1991). Compared to Mg salts, Ca(OH)_2_ is a widespread and low-cost material: costing approximately US$ 0.08 kg^−1^ while magnesium chloride costs about five times as much as Ca(OH)_2_ (Muster et al., 2013). Depending on where the Ca(OH)_2_ is sourced from and the quality of the reagent, the typical cost of the chemical per person per day would equate to approximately US$ 0.0008 (assuming a dosage of 10 g Ca(OH)_2_ L^−1^ of fresh urine and a person producing 1 L of fresh urine per day).

#### 4.3.3 Possible inactivation of pathogens

Literature shows that urine stabilization with the addition of Ca(OH)_2_ and the corresponding pH increase could lead to pathogen inactivation. Alkaline treatment is a well-known practice in wastewater treatment for pathogen inactivation (EPA, 1978, EPA, 2000, Girovich, 1996). Farrell et al. (1974) showed that the presence of bacteria in wastewater sludge can be reduced to a negligible level at a pH higher than 11.5, with a recommended exposure of 0.5 h and more. The observed pathogen removal was even higher at pH values of 12.5, leading to a reduction of the required exposure time. At pH values of 11.5, complete inactivation of viruses in soil at an exposure time of less than 1 h was shown by Pancorbo et al. (1988). However, for the inactivation of parasitic nematode worms such as Ascaris, pH values higher than 12 and exposure to this highly alkaline environment for several months are required (Eriksen et al., 1996). While these literature reference suggest that the pH values in stabilized urine will at least kill viruses and pathogens, further investigations are needed to determine the potential of Ca(OH)_2_ for disinfection.

## 5 Conclusions

We propose dosing Ca(OH)_2_ as a new method for stabilizing fresh urine because (a) no active dosing is needed, (b) the resulting saturation pH is in an operating region where neither enzymatic or chemical urea hydrolysis is relevant and (c) bacteria and viruses are killed at these high pH values as previous literature suggests. We have shown that dosing with Ca(OH)_2_ can also be used to precipitate calcium phosphate out of fresh urine as indicated by the decrease in dissolved phosphorus after the stabilization process. We also recommend providing a dosage of 10 g Ca(OH)_2_ L^−1^ of fresh urine. With this high dosage, saturation will be ensured for a wide range of urine compositions and operating conditions. Furthermore, we tentatively recommend an operating temperature range of 14 °C–40 °C to limit chemical urea hydrolysis.

## Figures and Tables

**Table 1:** Chemical formula of solid compounds used for chemical speciation calculations along with the name used in the PHREEQC database. Compounds with no name are described using the chemical formula only.

Formula	Name	Formula	Name	Formula	Name	Formula	Name
NH_4_Cl	–	CaCl_2_·6H_2_O	–	K_2_SO_4_	arcanite	Mg(OH)_2_	brucite
(NH_4_)_2_SO_4_	–	Ca_2_Cl_2_(OH)_2_·H_2_O	–	KHSO_4_	mercallite	MgOHCl	–
NH_4_MgPO_4_·6H_2_O	struvite	Ca_2_Cl_2_(OH)6·13H_2_O	–	K_2_HPO_4_		MgCl_2_	chloromagnesite
CaO	lime	NaCl	halite	KH_2_PO_4_		MgCl_2_·H_2_O	–
Ca(OH)_2_	portlandite	NaOH	–	K_3_H(SO_4_)_2_		MgCl_2_·2H_2_O	–
CaCl_2_	hydrophilite	Na_2_SO_4_	thenardite	K_8_H_6_(SO_4_)_7_	misenite	MgCl_2_·4H_2_O	–
CaSO_4_	anhydrite	Na_2_SO_4_·10H_2_O	mirabilite	KMgCl_3_·2H_2_O		MgCl_2_·6H_2_O	–
CaSO_4_·0.5H_2_O	hemihydrate	Na_3_H(SO_4_)_2_	–	KMgCl_3_·6H_2_O	carnallite	MgSO_4_	–
CaSO_4_·2H_2_O	gypsum	Na_2_Ca(SO_4_)_2_	glauberite	KMgClSO_4_·3H_2_O	kainite	MgSO4·H_2_O	kieserite
CaHPO_4_	DCPA	NaK_3_(SO_4_)_2_	glaserite	K_2_Mg_2_(SO_4_)_3_	langbeinit	MgSO_4_·4H_2_O	leonhardite
CaHPO_4_·2H_2_O	brushite	Na_2_Mg(SO_4_)_2_·4H_2_O	bloedite	K_2_Mg(SO_4_)_2_·4H_2_O	leonite	MgSO_4_·5H_2_O	pentahydrite
Ca_3_(PO_4_)_2_	TCP	Na_4_Ca(SO_4_)_3_·2H_2_O	labile	K_2_Mg(SO_4_)_2_·6H_2_O	shoenite	MgSO_4_·6H_2_O	hexahydrite
Ca_4_H(PO_4_)_3_	OCP	Na_2_HPO_4_	–	K_2_Ca(SO4)_2_·H_2_O	syngenite	MgSO_4_·7H_2_O	epsomite
Ca_5_(OH)(PO_4_)_3_	HAP	KCl	sylvite	K_2_Ca_5_(SO_4_)_6_·H_2_O	pentasalt	Mg_2_Cl(OH)_3_·4H_2_O	oxchloride-Mg
CaCl_2_·2H_2_O	–	KOH	–	K_2_MgCa_2_(SO_4_)_4_·2H_2_O	polyhalite	Mg_2_CaCl_6_·12H_2_O	tachyhydrite
CaCl_2_·4H_2_O	–	K_3_PO_4_		MgO	periclase		

**Table 2:** Composition of different fresh urine samples. U1a is the fresh urine before stabilization with Ca(OH)_2_ while U1b is the composition of the same urine after stabilization. The compositions U1, U2, U3 and U4 were measured in this study while U5 (Udert et al., 2006) and U6 (Udert et al., 2003b) were taken from literature. The composition of U5 is based on medical values. The columns marked with (sim.) for U1a and U1b show the values used for PHREEQC simulations. All other compositions were added as is into PHREEQC.

Measurement	Unit	U1a		U1b		U2	U3	U4	U5	U6
(meas.)	(sim.)	(meas.)	(sim.)
TIC	g m^−3^	28	–	33	–	–	–	–	–	–
Urea-N	g m^−3^	5420	–	5460	–	–	–	–	–	–
NO_2_-N	g m^−3^	<10	–	<10	–	–	–	–	–	–
NO_3_-N	g m^−3^	11	–	10	–	–	–	–	–	–
PO_4_-P	g m^−3^	260	260	<10	0	300	394	329	740	559
Total ammonia-N	g m^−3^	436	436	419	437	346	418	170	480	386
dissolved COD	g m^−3^	6400	6400	5800	6400	5900	6910	5500	10000	9700
Cl^−^	g m^−3^	4430	4430	4420	4420	4780	4790	3380	3800	5230
SO_4_	g m^−3^	825	825	812	823	825	817	673	1050	1500
Na^+^	g m^−3^	2510	5320	2400	5310	2600	2580	2340	2600	3730
K^+^	g m^−3^	469	469	348	469	2130	2810	2730	2200	2250
Ca^2+^	g m^−3^	132	132	1150	1900	100	326	77	190	168
Mg^2+^	g m^−3^	57	57	1	0	158	148	44	100	121
pH	–	6.3	6.3	12.6	12.6	6.6	6.88	6.67	6.2	6.0
